# Why Glucagon Matters for Hypoglycemia and Physical Activity in Individuals With Type 1 Diabetes

**DOI:** 10.3389/fcdhc.2022.889248

**Published:** 2022-07-08

**Authors:** Sheri R. Colberg

**Affiliations:** Human Movement Sciences Department, Old Dominion University, Norfolk, VA, United States

**Keywords:** glucagon, insulin, physical activity, type 1 diabetes, blood glucose, counterregulatory hormones, hypoglycemia, hyperglycemia

## Introduction

Type 1 diabetes (T1D) is an autoimmune disease that results in loss of insulin-making beta cells in the pancreas, which significantly affects the maintenance of blood glucose, both at rest and during physical activity. Anyone with this condition is challenged to keep blood glucose levels in normal ranges since all insulin must be supplied exogenously through subcutaneous injection of inhalation, and insulin levels that lower blood glucose must be balanced by the release of counterregulatory hormones that raise it. Exogenously administered insulin lasts in the body far longer than any that is endogenously released, which creates problems with adjusting for constantly changing insulin needs throughout the day. In addition, exogenous insulin comes in various types and formulations (ie, basal and mealtime), none of which can be withdrawn after administration or easily lowered for physical activity. Achieving glycemic balance during and following physical activity can be even more of a feat given that blood glucose use rises significantly during physical movement.

This hormonal balance is directly impacted by the interaction of counterregulatory hormones and insulin since the latter is not produced in and released from the pancreatic beta cells like normal in individuals with T1D. Most subcutaneously administered insulin fails to reach the liver, and administering insulin into the portal system through intraperitoneal delivery is not feasible at this time. While normally functioning beta cells make and release insulin, pancreatic alpha cells are responsible for the production of the hormone glucagon, the release of which occurs in response to hypoglycemia and is suppressed by elevations in blood glucose. In individuals with T1D, however, an altered ratio of glucagon-to-insulin at the liver alters its ability to manage the storage and release of glucose (1). It is my opinion that more effective balancing of these hormones is likely the most important blood glucose management step for individuals with T1D as long as exogenous insulin delivery *via* current methods remains the only option for its replacement. However, other pharmaceutical options to better manage glucagon release may also be on the horizon.

## Hepatic Management of Euglycemia

Why does where insulin is delivered matter? The liver is the key to maintaining blood glucose levels under a variety of physiological conditions, including physical activity (2). Postprandial hepatic glucose uptake is determined by splanchnic insulin levels and glucose elevations and delivery. In all individuals, liver glycogen stores are typically mostly depleted after an overnight fast and fully used after 36 to 48 hours of a fast, but hepatic gluconeogenesis is able to provide some additional glucose for the brain and nervous system once these glycogen stores are no longer available (3).

Hepatic glucose output increases during all physical activity, the rate of output determined by the intensity and duration of movement. Engaging in light- and moderate-intensity activities causes the release of only small amounts of glucose-raising hormones (unless extended in duration), but doing vigorous resistance training, sprint training, or high-intensity interval exercise results in an exaggerated hormonal response and excessive glucose production. During short, intensive bouts, rapid hepatic glycogenolysis is the primary source of extra glucose for working skeletal muscles, but this source becomes depleted during extended activities, making gluconeogenesis more important (4). However, gluconeogenesis is a slower process that tends to fail to keep up with high bodily demands for blood glucose during physical activity.

### Actions of Counterregulatory Hormones and Insulin

The actions of the five counterregulatory hormones that can raise blood glucose (directly or indirectly) are regulated by a variety of metabolic stressors like physical activity, overnight or extended fasting, emotional states, and illness (see **Table 1**). The fact that insulin alone lowers blood glucose while five hormones raise it shows a hormonal redundancy that proves that, at least from a survival standpoint, an adequate supply of blood glucose is critical to maintain. Insulin regulates the storage of macronutrients after meals and stimulates the cells in the body that are sensitive to it (skeletal muscle, adipose, and hepatic) to take up glucose to store as glycogen and/or triglycerides. The combined signal of high insulin and elevations in blood glucose cause the liver to store glucose as glycogen for later release, whereas an overnight fast or long-duration physical activities raise glucagon and drive glucose release. With the exception of amylin that is co-released with insulin, all other hormones described in **Table 1** are released in varying amounts based on the intensity and duration of physical movement or in response to hypoglycemia, all with the purpose of maintaining blood glucose through various and redundant metabolic pathways. During exercise, circulating insulin stimulates muscle glucose uptake such that in individuals without diabetes, insulin typically decreases and glucagon and other counterregulatory hormones rise (1).

**Table 1:** Counterregulatory hormones and Their Impact on Blood Glucose.

Hormone	Source	Main Actions
Insulin	Beta cells (pancreas)	Blood glucose uptake in insulin-sensitive muscle and adipose tissues; uptake and storage of glucose in the liver; inhibition of triglyceride release from adipose
Amylin	Beta cells (pancreas); normally co-secreted with insulin	Slower digestion and absorption of glucose from food; blocked glucagon release; early satiety
Glucagon	Alpha cells (pancreas)	Liver glycogenolysis and gluconeogenesis from precursors
Epinephrine (Adrenaline)	Adrenal medulla	Muscle and some liver glycogen breakdown; free fatty acid mobilization from adipose; insulin resistance in muscle
Norepinephrine	Adrenal medulla, sympathetic nerve endings	Hepatic gluconeogenesis; feed-forward control of glucose during vigorous activities (with epinephrine)
Growth hormone	Anterior pituitary	Release of free fatty acids from adipose; indirect suppression of blood glucose use; amino acid storage in muscles during rest
Cortisol	Adrenal cortex	Mobilization of amino acids and glycerol as precursors for hepatic gluconeogenesis; mobilization of free fatty acids from adipose for muscular use; insulin resistance

### Why Hypoglycemia Occurs in Diabetes

In individuals with T1D, the hormones that prevent hypoglycemia are typically impaired by the loss of glucagon release and a lesser release of epinephrine in response to declining levels of glucose in the blood (5). Why do these alterations occur? Insulin and glucagon levels at the liver are abnormal because the delivery of insulin is altered. At present, exogenous insulin must be injected under the skin, supplied using a pump *via* a subcutaneous catheter, or inhaled; all of these delivery methods lead to higher levels in the periphery and lower levels than normal in the portal circulation. Exercising with high peripheral levels of insulin raises hypoglycemia risk while lowering blood insulin helps normalize hormonal responses (6).

## Role of Glucagon in Hypoglycemia

In response to hypoglycemia, glucagon release may be blunted or absent in individuals with diabetes (7–10). This release is frequently lost early in the course of the disease (11), resulting in diminished glycogen stores in the liver, although glycogen storage in individuals with well-managed glycemia may not be impacted as much (12). When hepatic glycogen stores are diminished, gluconeogenesis is relied on to raise glucose output, and the driver of this hepatic process is also the release of glucagon, which is regulated by a variety of factors (13). Even when youth develop T1D, their glucagon release in response to hypoglycemia appears to be altered early after onset (the first year), and adults with T1D who have continued to make small amounts of their own insulin also have impaired and/or absent glucagon release when hypoglycemia occurs (14). The abnormal release of glucagon appears to be specific to hypoglycemia, however, since other conditions (like giving amino acids or withdrawing insulin and even physical activity itself) result in normal elevations of this hormone.

To compound the problem, apparently even having one bout of hypoglycemia or undertaking a single workout can result in a subnormal release of counterregulatory hormones (including glucagon and epinephrine) when an individual either experiences hypoglycemia or exercises later in the same day or on the next one (15–17). Even in adults without diabetes, hypoglycemia may blunt the overall hormonal response the next time it occurs to a certain extent, but their release of glucagon is not impacted (18). Although falling blood glucose levels would usually lead to an increase in hepatic glucose output, this process short-circuits when glucagon responses are diminished or absent, leading to hypoglycemia during exercise.

Conversely, impaired suppression of glucagon is uniquely associated with the portal insulin deficiency that occurs with exogenous insulin delivery in T1D (19), leading to hyperglycemia due to paradoxical hyperglucagonemia at other times, such as during overnight fasting and after meals (1). In fact, most with T1D experience an excessive output of glucose from the liver following meals and during the overnight hours.

In ferreting out the importance of the glucagon response in individuals with T1D, a recent mini-review (20) brought up a variety of points that should be considered:

* How the release of glucagon from the alpha cells of the pancreas is triggered is likely a complex process that involves some feedback loops and interactions with other hormones.

* A variety of factors can trigger glucagon release, including circulating amino acids, levels of blood glucose at the level of the pancreas, actions of the central nervous system, the release of gut incretins, and additional signals from beta and/or delta cells in the pancreas.

* When hypoglycemia occurs (either at rest or during physical activity), the release of glucagon is the key signal for the liver to either break down stored glycogen or use the process of gluconeogenesis to make and release glucose into the blood. Other counterregulatory hormones act to increase insulin resistance to slow the use of glucose and to provide metabolic precursors like lactate and alanine for new glucose production.

* The alpha cells of the pancreas respond to a much lesser extent when blood glucose levels decrease than they do to select amino acids like alanine, which gets released from working skeletal muscle during exercise and can be used as a precursor for gluconeogenesis.

* Even when glucagon release is absent in response to falling blood glucose levels, it is still normally released in response to many other changing metabolic conditions, such as periods of fasting, physical activity, and stress (emotional or physical); it appears that the triggers may differ for these stimuli compared to hypoglycemia.

* The cause of deficient or nonexistent release of glucagon in response to hypoglycemia in individuals with T1D relates to something that is within the islet cells of the pancreas.

* Excessive amounts of glucagon are frequently released in individuals with T1D after meals, and these higher levels can cause hyperglycemia after eating and insulin resistance.

### Role of Glucokinase in Managing Liver Glycogen

Perhaps another cause of glucagon dysfunction lies in the regulation of an enzyme in liver cells, glucokinase. What does glucokinase do, and why does it matter? When activated in the liver, glucokinase causes glucose that enters the liver *via* the circulation to be trapped there and stored as glycogen (4, 21). When activated in the pancreas, this enzyme serves as a glucose sensor in beta cells, and elevations in glucose in those cells lead to the secretion of insulin from fully functional beta cells (22), which clearly does not occur when beta cells are absent or destroyed in individuals with T1D. When glucose is detected in the alpha cells, glucagon released is blunted—but this process also requires activated glucokinase (19). When glucokinase remains inactive due to a lack of appropriate triggers, excessive amounts of glucagon are released and lead to elevations in blood glucose, even after eating when blood glucose levels may already be elevated.

How does this lack of glucokinase activation impact physical activity responses? When activated glucokinase in hepatic tissues has stimulated normal glycogen storage prior to activities, elevations in glucagon related to exercise (which are not impaired in T1D) can lead to more normal production of blood glucose from glycogen breakdown, leading to a lesser risk of hypoglycemia when active and even potentially for hours after exercise (13, 21). Having activated glucokinase in the alpha cells of the pancreas also blunts most glucose elevations that occur in the morning after an overnight fast in individuals with T1D.

### Role of Somatostatin in Glucagon Release

Another possible explanation of this diminished counterregulatory response to hypoglycemia in T1D may be the baseline hypersecretion of another hormone, somatostatin, and/or a greater activity of its receptors (23); such changes in somatostatin may also assist in explaining why even more glucagon is released when blood glucose levels are already elevated. Somatostatin is produced and released by the delta cells of the pancreas (24). Its release blocks insulin and glucagon from being released from their respective nearby pancreatic cells. ATP-sensitive potassium channels in the delta cells open when glucose levels are low and close when elevated (24). In animal models of diabetes, the secretion of somatostatin has been shown to be altered. When glucagon fails to be released by the stimulus of hypoglycemia, this defect may be corrected by activation of somatostatin receptor-2 antagonists. Some researchers are considering the impact of somatostatin antagonists or agents that suppress somatostatin secretion that could be used along with insulin therapy to restore a more appropriate release of glucagon during hypoglycemia and postprandial or fasting conditions (24).

## Discussion/Possible Solutions

Given the immediate medical crisis that is created by hypoglycemia, losing the ability to secrete normal amounts of glucagon to counteract it is one of the most critical problems to address in the effective management of T1D (5). Although exogenous insulin for the treatment of T1D has now been possible for over a century (25), achieving normoglycemia remains a challenge that is impacted more directly by the manner in which insulin is replaced than any other factor. The resulting loss of normal glucagon and other counterregulatory hormone release impacts glycemic regulation during rest and especially during physical activity when blood glucose levels decline. Given the current insulin delivery options, the best that most individuals with T1D can hope to achieve is as near to normal glucose levels as possible for a majority of the time (i.e., increased time-in-range). If a treatment for T1D can restore normal insulin release into the portal vein, glucose regulation and hypoglycemia prevention will certainly become easier during rest and activities.

While waiting for a more effective treatment or a diabetes cure, how can individuals with T1D make up for diminished or absent glucagon release during hypoglycemia? In some instances, undertaking a sprint or high-intensity interval during activities may cause a more exaggerated release of glucagon and epinephrine and raise blood glucose during more prolonged activities (26, 27). Also, taking in some amino acids (via a protein source) may improve the release of glucagon and cognition during bouts of hypoglycemia (28). Glucagon pens (which would be like an insulin pen but with glucagon delivered) that would allow mini-dosing with glucagon may soon be possible as well (29). Whatever the method, individuals with T1D need alternate ways to raise glucagon (that have not been impacted by their condition) to better manage hypoglycemia as long as insulin delivery stays the same.

It may also be possible to target hepatic glucokinase with other drugs to manage glucagon release (30). For instance, an investigatory drug labeled TTP399 could be taken orally and would act on the liver to activate glucokinase, thereby potentially lowering postprandial and morning blood glucose levels in individuals with T1D. If approved, it would be the first oral pill to treat T1D. In the SimpliciT1 study that reports on this drug (31), TTP399 use lowered A1C levels, along with overall insulin requirements over 12 weeks. Study participants needed 11 percent less insulin for meals and spent two more hours each day with blood glucose in optimal ranges. A benefit over the use of medications like SGLT-2 inhibitors (used off-label by individuals with T1D to lower postprandial glucose spikes), this investigational medication is much less likely to result in diabetic ketoacidosis, even with less circulating insulin present and a lower risk of hypoglycemia.

Even more recently, researchers have investigated a novel somatostatin receptor-2 antagonist, ZT-01, which they have used to stimulate glucagon release in rats with diabetes. Since it was effective for restoring glucagon release in animals, it has been postulated that ZT-01 may be able to restore the absent or diminished glucagon responses to hypoglycemia in individuals with T1D (5), although this effect has yet to be fully studied in humans. While it would be ideal to fix the problem of altered glucagon release in T1D due to low levels of insulin in the portal circulation with exogenous delivery, any treatment that can potentially make glucagon respond more appropriately all the time in individuals with type 1 diabetes would indeed be welcome.

## Author Contributions

SC is the sole author of this opinion piece and is responsible and accountable for the content and preparation of this article in its entirety.

## Conflict of Interest

The author declares the absence of any commercial or financial relationships that could be construed as a potential conflict of interest.

## Publisher’s Note

All claims expressed in this article are solely those of the authors and do not necessarily represent those of their affiliated organizations, or those of the publisher, the editors and the reviewers. Any product that may be evaluated in this article, or claim that may be made by its manufacturer, is not guaranteed or endorsed by the publisher.

## References

[1] Goodwin ML. Blood Glucose Regulation During Prolonged, Submaximal, Continuous Exercise: A Guide for Clinicians. J Diabetes Sci Technol (2010) 4(3):694–705. doi: 10.1177/193229681000400325 20513337 PMC2901048

[2] Wahren J Ekberg K. Splanchnic Regulation of Glucose Production. Annu Rev Nutr (2007) 27:329–45. doi: 10.1146/annurev.nutr.27.061406.093806 17465853

[3] Gonzalez JT Betts JA. Dietary Sugars, Exercise and Hepatic Carbohydrate Metabolism. Proc Nutr Soc (2019) 78(2):246–56. doi: 10.1017/S0029665118002604 30348238

[4] Adeva-Andany MM González-Lucán M Donapetry-García C Fernández-Fernández C Ameneiros-Rodríguez E. Glycogen Metabolism in Humans. BBA Clin (2016) 5:85–100. doi: 10.1016/j.bbacli.2016.02.001 27051594 PMC4802397

[5] Farhat R Aiken J D'Souza NC Appadurai D Hull G Simonson E. ZT-01: A Novel Somatostatin Receptor 2 Antagonist for Restoring the Glucagon Response to Hypoglycaemia in Type 1 Diabetes. Diabetes Obes Metab (2022) 24(5):908–17. doi: 10.1111/dom.14652 35060297

[6] Riddell MC Scott SN Fournier PA Colberg SR Gallen IW Moser O. The Competitive Athlete With Type 1 Diabetes. Diabetologia (2020) 63(8):1475–90. doi: 10.1007/s00125-020-05183-8 PMC7351823 32533229

[7] Yosten GLC. Alpha Cell Dysfunction in Type 1 Diabetes. Peptides (2018) 100:54–60. doi: 10.1016/j.peptides.2017.12.001 29412832

[8] Diabetes Research in Children Network Study G Tsalikian E Tamborlane W Xing D DM B Mauras N. Blunted Counterregulatory Hormone Responses to Hypoglycemia in Young Children and Adolescents With Well-Controlled Type 1 Diabetes. Diabetes Care (2009) 32(11):1954–9. doi: 10.2337/dc08-2298 PMC2768200 19675205

[9] Cryer PE. Minireview: Glucagon in the Pathogenesis of Hypoglycemia and Hyperglycemia in Diabetes. Endocrinology (2012) 153(3):1039–48. doi: 10.1210/en.2011-1499 PMC3281526 22166985

[10] Cryer PE. Hypoglycemia-Associated Autonomic Failure in Diabetes. Handb Clin Neurol (2013) 117:295–307. doi: 10.1016/B978-0-444-53491-0.00023-7 24095133

[11] Siafarikas A Johnston RJ Bulsara MK O'Leary P Jones TW Davis EA. Early Loss of the Glucagon Response to Hypoglycemia in Adolescents With Type 1 Diabetes. Diabetes Care (2012) 35(8):1757–62. doi: 10.2337/dc11-2010 PMC3402257 22699295

[12] Bally L Buehler T Dokumaci AS Boesch C Stettler C. Hepatic and Intramyocellular Glycogen Stores in Adults With Type 1 Diabetes and Healthy Controls. Diabetes Res Clin Practice (2015) 109(1):e1–3. doi: 10.1016/j.diabres.2015.05.002 26013568

[13] Moede T Leibiger B Vaca Sanchez P Daré E Köhler M Muhandiramlage TP. Glucokinase Intrinsically Regulates Glucose Sensing and Glucagon Secretion in Pancreatic Alpha Cells. Sci Rep (2020) 10(1):20145. doi: 10.1038/s41598-020-76863-z 33214580 PMC7678872

[14] Sherr J Xing D Ruedy KJ Beck RW Kollman C Buckingham B. Lack of Association Between Residual Insulin Production and Glucagon Response to Hypoglycemia in Youth With Short Duration of Type 1 Diabetes. Diabetes Care (2013) 36(6):1470–6. doi: 10.2337/dc12-1697 PMC3661789 23288858

[15] Davis SN Mann S Galassetti P Neill RA Tate D Ertl AC. Effects of Differing Durations of Antecedent Hypoglycemia on Counterregulatory Responses to Subsequent Hypoglycemia in Normal Humans. Diabetes (2000) 49(11):1897–903. doi: 10.2337/diabetes.49.11.1897 11078457

[16] Galassetti P Tate D Neill RA Morrey S Wasserman DH Davis SN. Effect of Antecedent Hypoglycemia on Counterregulatory Responses to Subsequent Euglycemic Exercise in Type 1 Diabetes. Diabetes (2003) 52(7):1761–9. doi: 10.2337/diabetes.52.7.1761 12829644

[17] Sandoval DA Guy DL Richardson MA Ertl AC Davis SN. Acute, Same-Day Effects of Antecedent Exercise on Counterregulatory Responses to Subsequent Hypoglycemia in Type 1 Diabetes Mellitus. Am J Physiol Endocrinol Metab (2006) 290(6):E1331–8. doi: 10.1152/ajpendo.00283.2005 16449302

[18] Moheet A Kumar A Eberly LE Kim J Roberts R Seaquist ER. Hypoglycemia-Associated Autonomic Failure in Healthy Humans: Comparison of Two vs Three Periods of Hypoglycemia on Hypoglycemia-Induced Counterregulatory and Symptom Response 5 Days Later. J Clin Endocrinol Metab (2014) 99(2):664–70. doi: 10.1210/jc.2013-3493 PMC3913799 24423306

[19] Basco D Zhang Q Salehi A Tarasov A Dolci W Herrera P. α-Cell Glucokinase Suppresses Glucose-Regulated Glucagon Secretion. Nat Commun (2018) 9(1):546. doi: 10.1038/s41467-018-03034-0 29416045 PMC5803227

[20] Bisgaard Bengtsen M Møller N. Mini-Review: Glucagon Responses in Type 1 Diabetes - a Matter of Complexity. Physiol Rep (2021) 9(16):e15009. doi: 10.14814/phy2.15009 34405569 PMC8371343

[21] Matschinsky FM Wilson DF. The Central Role of Glucokinase in Glucose Homeostasis: A Perspective 50 Years After Demonstrating the Presence of the Enzyme in Islets of Langerhans. Front Physiol (2019) 10:148. doi: 10.3389/fphys.2019.00148 30949058 PMC6435959

[22] Toulis KA Nirantharakumar K Pourzitaki C Barnett AH Tahrani AA. Glucokinase Activators for Type 2 Diabetes: Challenges and Future Developments. Drugs (2020) 80(5):467–75. doi: 10.1007/s40265-020-01278-z 32162273

[23] Briant L Salehi A Vergari E Zhang Q Rorsman P. Glucagon Secretion From Pancreatic α-Cells. Ups J Med Sci (2016) 121(2):113–9. doi: 10.3109/03009734.2016.1156789 PMC4900066 27044683

[24] Rorsman P Huising MO. The Somatostatin-Secreting Pancreatic δ-Cell in Health and Disease. Nat Rev Endocrinol (2018) 14(7):404–14. doi: 10.1038/s41574-018-0020-6 PMC5997567 29773871

[25] Gerstein HC Rutty CJ. Insulin Therapy: The Discovery That Shaped a Century. Can J Diabetes (2021) 45(8):798–803. doi: 10.1016/j.jcjd.2021.03.002 34045148

[26] Bussau VA Ferreira LD Jones TW Fournier PA. A 10-s Sprint Performed Prior to Moderate-Intensity Exercise Prevents Early Post-Exercise Fall in Glycaemia in Individuals With Type 1 Diabetes. Diabetologia (2007) 50(9):1815–8. doi: 10.1007/s00125-007-0727-8 17583795

[27] Guelfi KJ Ratnam N Smythe GA Jones TW Fournier PA. Effect of Intermittent High-Intensity Compared With Continuous Moderate Exercise on Glucose Production and Utilization in Individuals With Type 1 Diabetes. Am J Physiol Endocrinol Metab (2007) 292(3):E865–70. doi: 10.1152/ajpendo.00533.2006 17339500

[28] Rossetti P Porcellati F Busciantella Ricci N Candeloro P Cioli P Nair KS. Effect of Oral Amino Acids on Counterregulatory Responses and Cognitive Function During Insulin-Induced Hypoglycemia in Nondiabetic and Type 1 Diabetic People. Diabetes (2008) 57(7):1905–17. doi: 10.2337/db08-0276 PMC2453632 18390791

[29] Haymond MW Redondo MJ McKay S Cummins MJ Newswanger B Kinzell J. Nonaqueous, Mini-Dose Glucagon for Treatment of Mild Hypoglycemia in Adults With Type 1 Diabetes: A Dose-Seeking Study. Diabetes Care (2016) 39(3):465–8. doi: 10.2337/dc15-2124 PMC4764034 26861921

[30] Bahl V Lee May C Perez A Glaser B Kaestner KH. Genetic Activation of α-Cell Glucokinase in Mice Causes Enhanced Glucose-Suppression of Glucagon Secretion During Normal and Diabetic States. Mol Metab (2021) 49:101193. doi: 10.1016/j.molmet.2021.101193 33610858 PMC7973249

[31] Klein KR Freeman JLR Dunn I Dvergsten C Kirkman MS Buse JB. The SimpliciT1 Study: A Randomized, Double-Blind, Placebo-Controlled Phase 1b/2 Adaptive Study of TTP399, a Hepatoselective Glucokinase Activator, for Adjunctive Treatment of Type 1 Diabetes. Diabetes Care (2021) 44(4):960–8. doi: 10.2337/dc20-2684 PMC7985421 33622669

